# Reliability of Peak Running Velocity Obtained on the Track Field in Runners of Different Performance Levels

**DOI:** 10.3389/fphys.2021.680913

**Published:** 2021-12-13

**Authors:** Francisco de Assis Manoel, Cecília Segabinazi Peserico, Fabiana Andrade Machado

**Affiliations:** ^1^Department of Physical Education, Cesumar University, Maringá, Brazil; ^2^Department of Physical Education, Federal University of Lavras, Lavras, Brazil; ^3^Department of Physical Education, State University of Maringá, Maringá, Brazil; ^4^Associate Post-graduate Program in Physical Education UEM/UEL, Department of Physical Education, State University of Maringá, Maringá, Brazil; ^5^Post-graduate Program of Physiological Sciences, Department of Physiological Sciences, State University of Maringá, Maringá, Brazil

**Keywords:** reproducibility, effort testing, athletic performance, exercise, training

## Abstract

The aim of this study was to verify the reliability of peak running velocity obtained on the track field (V_peak_TF_) in runners of different performance levels. 39 male endurance runners were divided into two groups: trained runners (TR; *n* = 22; 10-km time running performance of 35.2 ± 1.7 min), and recreational runners (RR; *n* = 17; 10-km time running performance of 51.3 ± 4.8 min). They performed three maximal incremental running tests on the official track field (400 m), with an interval of 1 week between trials to determine the reliability of V_peak_T_. The V_peak_TF_ showed high reliability, presenting an intraclass correlation coefficient and coefficient of variation of 0.97 and 1.28%, and 0.90 and 1.24% for TR and RR, respectively. Both TR and RR showed lowest bias and limits of agreement between test and retest (V_peak_TF1_ and V_peak_TF2_). In addition, there was no statistical test-retest difference for V_peak_TF_. In addition, the HR and RPE submaximal values were reliable for both TR and RR. Therefore, the V_peak_TF_ showed high reliability in both TR and RR. These findings reinforce that the protocol for determining V_peak_TF_, using increments of 1 km h^–1^ every 3 min is reliable regardless of the performance level of the runners.

## Introduction

Incremental test protocols have been used to determine aerobic parameters that are important to decide the appropriate prescription and monitoring the endurance training program (Buchheit et al., 2010; Manoel et al., 2017). However, the indexes determined during these tests must be reliable to ensure that individual measures are accurate and sensitive to detect changes caused by differences in the training period (Hopkins et al., 2011; Peserico et al., 2014).

Physiological indexes determined during incremental tests (e.g., lactate threshold – LT and maximal oxygen uptake – $\dot{\text{V}}$O_2max_) are already well documented in the literature as reliable for identifying exercise intensities (Billat and Koralsztein, 1996; Mclaughlin et al., 2010; Hopkins et al., 2011). Concerning the reliability of peak velocity (V_peak_), defined as the maximal velocity obtained in an incremental running test (Noakes et al., 1990; Machado et al., 2013), this variable was studied concerning its reliability only for the V_peak_ determined during treadmill protocols (V_peak_T_; Peserico et al., 2014; Cerezuela-Espejo et al., 2018).

Peserico et al. (2014) examined the test-retest reliability of V_peak_T_ obtained from three maximal incremental tests with different velocities increments (0.5, 1, and 2 km⋅h^–1^) and a fixed 3-min stages duration, and demonstrated high reliability of V_peak_T_, with low standard error of measurement (SEM; ≤0.3 km h^–1^), a coefficient of variation (CV) ≤1.8%; intraclass correlation coefficient (ICC) ≥0.90 in 31 recreational runners (RR). In addition, Cerezuela-Espejo et al. (2018) reported that V_peak_T_ comprising 1 min stage duration with increments of 1 km h^–1^ was reliable with low SEM (2.79 km h^–1^), low CV (3.1%) and ICC of 0.94 in 22 trained runners (TR).

Thus, although it has been shown that V_peak_T_ is reliable (Peserico et al., 2014; Cerezuela-Espejo et al., 2018), to the best of our knowledge, no study tested or investigated the reliability of V_peak_ obtained during a test performed on the track field (V_peak_TF_) using the same already-developed protocol for V_peak_T_ determination (Machado et al., 2013; Peserico et al., 2014).

Therefore, the aim of this study was to verify the reliability of V_peak_TF_ in runners of different performance levels. The hypothesis is that V_peak_TF_ is as reliable as the V_peak_T_, even for groups of runners of different performance levels.

## Materials and Methods

### Participants

In order to define the number of participants required for this study, *a priori*, calculation (*F* test; mixed measure of repeated measures between factors) was performed using the Gpower^®^ software, version 3.1, (Düsseldorf, Germany) according to an effect size (0.25), using V_peak_ and its correlation with 10-km running performance as outcome variables from a pilot study. With a power of 80% and an alpha of 0.05, the *a priori* analysis revealed a minimal sample of 34 participants.

Thirty-nine male endurance runners participated in this study and they were divided in two groups: 22 TR (Mean ± SD age: 30.6 ± 5.1 years, weight: 67.7 ± 9.7 kg, and height: 174.8 ± 4.2 cm) with a 10-km running times of 35.2 ± 1.7 min, [which represented ≅ 74.6% of the mean velocity (MV) of the World record]. They were experienced in competitive long-distance races with training frequency of 6 ± 1 days wk^–1^, and distance of 96.4 ± 23.4 km wk^–1^; 17 RR (Mean ± SD age: 29.2 ± 4.0 years, weight: 83.6 ± 9.9 kg, and height: 179.8 ± 8.5 cm), presented a 10-km running times 51.3 ± 4.8 min (which represented ≅ 51.1% of the MV of the World record). They were experienced in competitive long-distance races with training frequency of 3 ± 1 days wk^–1^, and distance of 24.5 ± 7.0 km wk^–1^.

All the participants presented medical clearance to perform exhaustive physical tests. They volunteered to participate in this study and were informed about the testing and possible risks involved and provided written informed consent. The experimental protocol was approved by the University’s Human Research Ethics Committee (#1.022.468/2015).

### Experimental Design

After being familiarized with the rating of perceived exertion (RPE) scale (Borg, 1982) and with the equipment to be used in the evaluations, the participants performed three maximal incremental running tests on the official track field (400 m) to determine the V_peak___TF_. The first test was used to adapt the participants to the protocol, being the second and third tests to verify test -retest reliability.

The tests were performed with an interval of 1 week between them, at the same time of the day (between 5:00 and 9:00 pm) under similar climatic conditions (temperature = 19–29^°^C and relative humidity = 56–72%). The total time, heart rate (HR), RPE and lactate concentrations were also obtained during these tests.

#### Determination of V_peak_ on the Track Field (V_peak_TF_)

After 3-min warm-up walking at 6 km h^–1^, the protocol started with an initial velocity of 8 km h^–1^, followed by an increase of 1 km h^–1^ every 3 min until volitional exhaustion (i.e., participant was unable to continue running; Machado et al., 2013). The velocity during the test was controlled by sound signals. Participants were instructed to cross the line of cones, which were distributed on the track field every 25 m, with at least one foot simultaneously to the beep (Léger and Boucher, 1980). The interval between the beeps at each stage decreased every 3 min, and the higher beep indicate that a new stage was starting, so the grader progressively increased the running velocity. The test was ended when was observed a decrease in speed due to fatigue (exhaustion) of the participant or when the evaluator identified that the runner failed to cross the cone line with one of two feet twice in a row (Léger and Boucher, 1980).

If the last stage was not completed, the V_peak_TF_ was calculated on the part-time remained in the last stage achieved from the equation proposed by Kuipers et al. (2003): V_peak_TF_ = V_complete_ + (Inc × *t*/*T*), in which V_complete_ is the running velocity of the last complete stage, Inc is the velocity increment (i.e., 1 km h^–1^), *t* is the number of seconds sustained during the incomplete stage, and *T* is the number of seconds required to complete a stage (i.e., 180 s).

#### Psychophysiological and Physiological Variables

During the incremental tests HR, RPE and lactate concentrations were collected. HR submaximal values were monitored during all tests (Polar^®^ RS800sd; Kempele, Finland) and HR_max_ was defined as the highest HR value recorded during the test. RPE submaximal values were also monitored during all tests by using a 6–20 Borg scale (Borg, 1982), and the highest RPE value was adopted as the peak RPE (RPE_max_). The reliability of the submaximal HR and RPE values were analyzed for TR and RR groups at the intensities of 8, 10, 12, and 14 km⋅h^–1^ because these velocities were the main and common to all runners; additionally, at 16 and 18 km⋅h^–1^ the reliability for HR and RPE were examined for TR because only this group reached these intensities.

Furthermore, we analyzed the reliability of submaximal velocities corresponding to low, moderate and high intensities based on% HR_max_ (Edwards, 1993). Thus, the reliability of velocities at 70% HR_max_ (i.e., low), 80%HR_max_ (i.e., moderate), and 90%HR_max_ (i.e., high) were demonstrated. The determination of submaximal velocities corresponding do% of HR_max_ were analyzed by linear interpolation considering the HR values and the speed above and below the HR values that represented 70, 80, and 90% of HR_max_.

For the lactate concentrations determination, earlobe capillary blood samples (25 μl) were collected into a capillary tube at the end of the tests (time zero of recovery) and at the third, fifth, and seventh minutes of passive recovery with participants seated in a comfortable chair. From these samples, lactate concentrations were subsequently determined by electroenzymatic methods using an automated analyzer (YSI 2300 STAT^®^, Yellow Springs, OH, United States). Peak lactate concentrations (Lac_peak_) were defined for each participant as the highest post-exercise lactate concentrations value.

### Statistical Analyses

Data were performed using the software Statistical Package for the Social Sciences (SPSS^®^ v.20, Inc., Chicago, IL, United States). The Shapiro–Wilk test was used to check the normality of the data being presented as mean ± SD. Hopkins spreadsheets were used to calculate reliability parameters; the reliability was considered high for ICC values of 0.90 greater, moderate for values between 0.80 and 0.89 and questionable for values below 0.80 (Vincent, 2005). The SEM and CV were calculated to represent the absolute reliability. The SEM were calculated by dividing the SD of the differences between the variables of the test and retest by the square root of two (√2; Hopkins et al., 2011). The CV was determined by obtaining the SEM of the natural logarithm of the variables (SEMln). Thereafter, the CV was calculated using the formula CV (%) = 100 × [exp (SEMln)–1], where exp is the natural exponential function. Test and retest of track field performances were compared by the *t*-test for paired samples. The Bland-Altman analysis was also used to calculate the bias (difference between the means) for the test-retest V_peak_TF_ with the respective limits of agreement (LoA) for a 95% interval. A significance level of *P* < 0.05 was adopted for all analyzes.

## Results

Table 1 shows the comparison between test and retest for V_peak_TF_, test duration, HR_max_, RPE_max_ and Lac_peak_ obtained in the incremental protocol for TR, RR and all runners. No significant differences were found between test and retest for all variables.

**TABLE 1:** Mean values ± standard deviation (SD) for the variables obtained during the maximal incremental tests on the track field for TR, RR, and all runners.

	TR (*n* = 22)			RR (*n* = 17)			All runners (*n* = 39)		
Variables	Test	Retest	*P*	Test	Retest	*P*	Test	Retest	*P*
V_peak_TF_ (km⋅h^–1^)	18.1 ± 1.2	18.0 ± 1.2	1.0	14.2 ± 0.6	14.3 ± 0.7	0.4	16.7 ± 1.8	16.4 ± 2.2	0.3
Duration (min)	36.1 ± 3.3	36.2 ± 3.1	0.9	25.0 ± 1.8	25.5 ± 2.0	0.2	30.9 ± 6.5	31.1 ± 6.4	0.2
HR_max_ (bpm)	182.0 ± 11.2	183.0 ± 9.7	0.1	188.0 ± 8.6	187.0 ± 11.1	0.3	180.0 ± 28.4	180.0 ± 28.5	0.6
RPE_max_ (AU)	19.9 ± 0.4	19.9 ± 0.3	1.0	19.6 ± 0.8	19.8 ± 0.7	1.0	19.7 ± 0.7	19.7 ± 0.8	1.0
Lac_peak_ (mmol⋅L^–1^)	7.8 ± 2.4	8.0 ± 1.8	0.6	8.4 ± 2.3	8.3 ± 2.6	0.69	8.3 ± 2.7	8.3 ± 2.5	0.9

*Note: TR, trained runners; RR, recreational runners; V_peak_TF_, peak velocity on track field; HR_max_, maximum heart rate; RPE_max_, subjective perception of maximal effort; and Lac_peak_, lactate peak.*

*P > 0.05.*

Table 2 demonstrate the test-retest reliability of V_peak_TF_ for TR, RR and all runners. V_peak_TF_ showed high reliability for both groups, due to the high ICC and low SEM and CV values.

**TABLE 2:** Reliability of V_peak_ on track field (V_peak___TF_) for trained runners (TR), recreational runners (RR), and all runners.

Groups	ICC (CI 95%)	SEM (km⋅h^–1^; CI 95%)	CV (%; CI 95%)
TR	0.97 (0.92–0.99)	0.22 (0.17–0.32)	1.28 (0.96–1.89)
RR	0.90 (0.75–0.96)	0.22 (0.16–0.33)	1.54 (0.14–2.35)
All runners	0.99 (0.98–0.99)	0.22 (0.18–0.29)	1.28 (0.98–1.83)

*Note: TR, trained runners; RR, recreational runners; ICC, intraclass correlation coefficient; SEM, standard error of measurement; CV, coefficient of variation; CI, confidence interval.*

**FIGURE 1:**
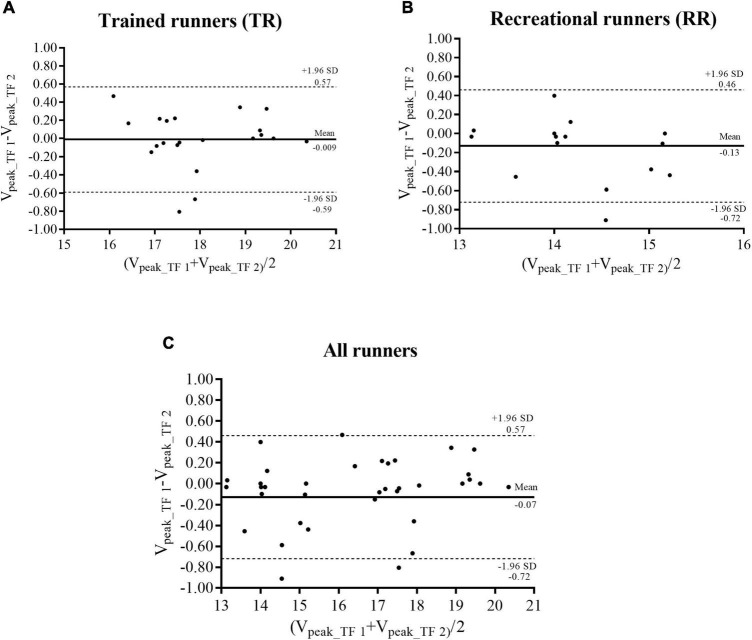
Bland-Altman plot of V_peak___TF1_ vs., V_peak_TF2_, for trained runners (TR; **A)**, recreational runners (RR; **B)**, and all runners **(C)**. Dotted lines represent the bias and solid lines denote lower and upper 95% limits of agreement.

Figure 1 shows the Bland-Altman analysis for the agreement between test (V_peak_TF1_) and retest (V_peak___TF2_). It was observed a low mean bias (<0.20 km h^–1^) for TR, RR and all runners; narrowest LoA for TR (−0.59 to 0.57 km⋅h^–1^), RR (0.72–0.46 km⋅h^–1^), and for all runners (0.72 to 0.57 km⋅h^–1^) were obtained.

Table 3 presents the reliability of submaximal intensities, obtained during the V_peak___TF_ test, corresponding to low, moderate, and high based on 70% HR_max,_ 80% HR_max,_ and 90% HR_max_, respectively, for TR and RR. No significant differences were found between test and retest for all submaximal velocities. For both groups of runners, the intensities were high reliable, mainly due the high ICC and low SEM values.

**TABLE 3:** Reliability of the submaximal intensities corresponding to low (70%HR_max_), moderate (80%HR_max_), and high intensities (90%HR_max_) obtained during the test to V_peak___TF_ for trained runners (TR) and recreational runners (RR).

Intensities	Groups	Test (km⋅h^–1^)	Retest (km⋅h^–1^)	ICC (CI 95%)	SEM (CI 95%)	CV (%; CI 95%)
Low	TR	9.8 ± 1.6	9.8 ± 1.6	0.95 (0.88–0.98)	0.37 (0.29–0.53)	3.83 (2.88–5.72)
	RR	8.0 ± 0.8	7.9 ± 0.7	0.84 (0.61–0.94)	0.33 (0.24–0.50)	4.01 (2.97–6.17)
Moderate	TR	11.9 ± 1.3	11.9 ± 1.6	0.93 (0.84–0.97)	0.41 (0.31–0.58)	4.11 (3.09–6.14)
	RR	9.7 ± 1.0	9.6 ± 0.9	0.85 (0.63–0.94)	0.39 (0.29–0.60)	4.06 (3.01–6.24)
High	TR	14.6 ± 1.2	14.5 ± 1.3	0.93 (0.85–0.97)	0.31 (0.57–1.05)	2.14 (1.61–3.18)
	RR	12.0 ± 1.3	12.0 ± 1.1	0.83 (0.59–0.94)	0.36 (0.27–0.54)	2.96 (2.19–4.54)

*Note: TR, trained runners; RR, recreational runners; ICC, intraclass correlation coefficient; SEM, standard error of measurement; CV, coefficient of variation; and CI, confidence interval.*

The test-retest reliability of HR and RPE values at submaximal intensities for TR and RR are presented in Tables 4, 5, respectively. The comparison between test and retest showed that all variables did not significantly differ in the TR group (Table 3); for RR, only HR values at 6, 12, and 14 km⋅h^–1^ differs between test and retest (Table 5). For both, TR and RR, the ICC were high (HR = 0.91 to 0.99; RPE = 0.71 to 0.95), the SEM ranged from 1.68 to 4.46 bpm for HR values and ranged from 0.30 to 1.05 for RPE values. The CV indices varied among the different intensities (HR = 1.02 to 3.33%; RPE = 3.98 to 11.04%).

**TABLE 4:** Reliability of the submaximal HR and RPE values obtained during the test to V_peak___TF_ for trained runners (TR).

Velocities (km⋅h^–1^)	Variables	Test	Retest	ICC (CI 95%)	SEM (CI 95%)	CV (%; CI 95%)
8	HR	119.0 ± 13.8	119.0 ± 13.2	0.94 (0.85–0.97)	3.64 (2.80–5.21)	3.20(2.41-4.77)
	RPE	7.32 ± 0.8	7.32 ± 0.7	0.71 (0.42–0.87)	0.41 (0.31–0.58)	5.47(4.11-8.20)
10	HR	132.0 ± 13.8	132.0 ± 15.1	0.92 (0.82–0.97)	4.46 (3.43–6.37)	3.28(2.52-4.72)
	RPE	8.1 ± 1.6	8.3 ± 1.4	0.81 (0.59–0.91)	0.72 (0.55–1.03)	9.07(6.91-13.21)
12	HR	147.0 ± 13.6	148.0 ± 13.6	0.91 (0.80–0.96)	4.31 (3.31–6.16)	2.93(2.21-4.36)
	RPE	10.0 ± 2.3	10.0 ± 2.1	0.84 (0.66–0.93)	0.97 (0.75–1.38)	11.04(8.24-16.76)
14	HR	161.0 ± 11.4	161.0 ± 11.7	0.93 (0.84–0.97)	3.18 (2.44–4.54)	2.04(1.54-3.04)
	RPE	12.3 ± 2.4	12.3 ± 2.3	0.90 (0.77–0.96)	0.80 (0.61–1.14)	7.31(5.47-11.00)
16	HR	173.0 ± 10.6	174.0 ± 10.9	0.92 (0.81–0.96)	3.19 (2.45–4.55)	1.81(1.36-2.68)
	RPE	14.9 ± 2.7	15.2 ± 2.6	0.86 (0.69–0.94)	1.05 (0.81–1.50)	8.59(6.42-12.96)
18	HR	179.0 ± 10.3	180.0 ± 10.9	0.94 (0.76–0.99)	3.05 (2.06–5.84)	1.74(1.18-3.37)
	RPE	17.3 ± 2.9	17.6 ± 2.7	0.95 (0.81–0.99)	0.72 (0.48–1.37)	4.85(3.25-9.49)

*Note: TR, trained runners; HR, heart rate; RPE, rating of perceived exertion; ICC, intraclass correlation coefficient; SEM, standard error of measurement; CV, coefficient of variation; and CI, confidence interval.*

**TABLE 5:** Reliability of the submaximal HR and RPE values obtained during the test to determine V_peak___TF_ for recreational runners (RR).

Velocities (km⋅h^–1^)	Variables	Test	Retest	ICC (CI 95%)	SEM (CI 95%)	CV (%; CI 95%)
8	HR	132.0 ± 13.8	131 ± 15.5	0.93 (0.82–0.97)	4.13 (3.07–6.28)	3.33(2.47-5.12)
	RPE	7.3 ± 0.6	7.4 ± 0.7	0.80 (0.54–0.92)	0.30 (0.23–0.46)	3.98(2.95-6.12)
10	HR	153.0 ± 13.1	153.0 ± 13.3	0.97 (0.91–0.99)	2.59 (1.93–3.93)	1.72(1.28-2.63)
	RPE	9.8 ± 2.4	9.8 ± 2.3	0.93 (0.82–0.97)	0.66 (0.49–1.01)	6.56(4.85-10.16)
12	HR	173.0 ± 12.5	171.0 ± 13.0*	0.99 (0.96–0.99)	1.68 (1.25–2.55)	1.02(0.72-1.56)
	RPE	13.4 ± 3.1	13.2 ± 3.4	0.93 (0.82–0.97)	0.92 (0.68–1.40)	8.93(6.58-13.90)
14	HR	184.0 ± 11.2	182.0 ± 12.2*	0.92 (0.76–0.97)	3.10 (2.24–4.99)	1.80(1.30-2.92)
	RPE	18.2 ± 2.3	17.8 ± 2.8	0.95 (0.85–0.98)	0.66 (0.48–1.06)	4.77(3.43-7.79)

*Note: RR, recreational runners; HR, heart rate; RPE, rating of perceived exertion; ICC, intraclass correlation coefficient; SEM, standard error of measurement; CV, coefficient of variation; and CI, confidence interval.*

*P < 0.05 compared to test.

Concerning the reliability of HR_max_ and RPE_max_, it was not found significant differences between test and retest. The reliability measures indicate high reliability for both TR and RR (ICC: HR_max_ = 0.90 and 0.88; RPE_max_ = 1.00; SEM: HR_max_ 2.07 and 3.66; RPE_max_ = 0.00; and CV: HR_max_ = 2.07 and 2.11%; and RPE_max_ = 0.00). Additionally, the Lac_peak_ values were not significant different between tests and retest. The reliability of Lac_peak_, for both TR and RR, presented high ICC values (0.81 and 0.88), low SEM (1.0 and 0.88 mmol⋅L^–1^), and high CV indices (16.53 and 14.87%).

## Discussion

The aim of this study was to verify the reliability of V_peak_TF_ in runners of different performance levels. The main finding was that V_peak_TF_ showed high reliability for TR and RR. In addition, the maximum and submaximal indices determined during the test were reliable, showing similar responses for both groups. Thus, confirming our hypothesis.

The reliability of a test or a variable is an important construct, as it ensures consistency of the measurements obtained at different times throughout an assessment (Atkinson and Nevill, 1988; Weir, 2005; Hopkins et al., 2011). For demonstrating that the test or variable is reliable, some statistical measurements are calculated, in which the analyses need to present high ICC, low CV and SEM, and the LoA obtained by the Bland-Altman method must be equally low (Bland and Altman, 1986; Atkinson and Nevill, 1988; Currell and Jeukendrup, 2008).

However, the “acceptable” values of each measurement when performing an analysis of reliability are not established in the literature; hence, it is challenging to consider the variable reliable, as these values can change from variable to variable. In the present study, V_peak_TF_ showed high test-retest reliability in the TR and RR, with high ICC (0.97 and 0.90, respectively), lowest SEM (0.22 and 0.22 km⋅h^–1^, respectively) and CV (1.28 and 1.54%, respectively). These results are similar for V_peak_T_ obtained in previous studies with runners of different performance levels (Peserico et al., 2014; Cerezuela-Espejo et al., 2018).

Peserico et al. (2014) verified the test-retest reliability of V_peak_T_ and physiological variables during three maximum incremental tests; the authors found that the V_peak_T_, determined from the same incremental protocol used in the present study for V_peak_TF_ determination, was reliable. The following reliability values obtained were: ICC = 0.91, SEM = 0.3 km⋅h^–1^ and CV = 1.7%. Cerezuela-Espejo et al. (2018) after verifying the reliability of V_peak_T_ obtained during an incremental test using gas analyzer and with lactate sampling, observed an ICC value of 0.94 and CV of 2.79% after evaluating well-TR, which was similar to our findings concerning the V_peak_TF_.

As a complementary analysis, the Bland-Altman for V_peak_TF_ between test and retest demonstrated high agreement with lowest mean bias and narrowest LoA for both groups of runners. This result is important because it suggests that there was good agreement for V_peak_TF_ when evaluated under repeated conditions; furthermore as V_peak_TF_ will be used to prescribe and monitor the effects of training, it is important to confirm that the possible changes in V_peak___TF_ values occur due the training and not by the method used to evaluate the variable Similar to our results, Cerezuela-Espejo et al. (2018) verified the reliability of V_peak_T_ and found a bias of 0.12 km⋅h^–1^ and LoA between −0.71 to 0.95 km⋅h^–1^. These results reinforce the reliability of the protocol to determine V_peak_TF_ in the present study.

Concerning the reliability of the submaximal intensities analyzed (Table 3) and submaximal HR and RPE values (Tables 4, 5), it was demonstrated that the incremental protocol to determine V_peak_TF_ was reliable during all test (submaximal and maximal intensity); in addition, TR and RR groups had similar responses in relation to the reliability measures (e.g., ICC, SEM, and CV). Our results showed high reliability for HR values (ICC > 0.91; SEM < 4.46 bpm; and CV < 3.33%), that was similar to previous studies with runners who performed incremental tests on treadmill (test-retest; Lourenço et al., 2011; Peserico et al., 2015; Blagrove et al., 2017). Peserico et al. (2015) examined the same submaximal intensities run by runners in our study (8, 10, 12, and 14 km⋅h^–1^) and found high reliability (ICC = 0.47–0.79; SEM = 3.0–7.5 bpm; and CV = 1.5–5.8%) for HR during the intermediate-increment protocol (i.e., 3 min stages and increments of 1 km⋅h^–1^). Lourenço et al. (2011) reported a CV of 4.3% and a SEM of 6.2 bpm for the reliability of HR at ventilatory threshold and Blagrove et al. (2017) also found lower variability for HR values at submaximal intensities (mean difference: 4 ± 4 bpm; ICC > 0.86).

The HR values (ICC > 0.71; SEM < 1.05; and CV < 11.04%) at submaximal intensities presented better reliable parameters compared to the RPE values. Peserico et al. (2015) verified limited reliability for the RPE values (i.e., intermediate-increment protocol) with lower ICC and higher indices of SEM and CV compared to those obtained in our study (ICC = 0.09–0.75; SEM = 0.8–1.4; and CV = 6.9–15.3%). In this same line Grant et al. (2002) examined the reliability of the RPE corresponding to the lactate threshold and reported that the use of RPE to prescribe intensity at lactate threshold has severe limitations.

In the present study, no difference was observed concerning the reliability in runners of different levels of performance. Different from our findings, previous studies reported that the level of performance has an impact on the results of the reliability of a test, in which more trained, experienced and faster runners tend to be more reliable (i.e., less variable) compared to younger or less experienced runners (Schabort et al., 1998; Hopkins et al., 2001). In addition, the CV of non-athletes is 1.3 times greater than that of athletes (Hopkins et al., 2001). The absence of difference in the runners’ reliability values in the present study can be justified by the fact that even the athletes present different levels of performance, both had an experience in running training and road and track field run competitions.

Despite the important findings, this study had some limitations such as the lack of a third test (e.g., trial) that could provide a more accurate impression for V_peak_TF_, and the lack of a dietary recall to control and standardize the same diet before the testing sessions; however, it is important to note that we recommended for the participants to maintain the same diet pattern before each test. Other limitation was the absence of another test using the gas analyzer to obtain ventilatory parameters; however, future studies can investigate the relationship between V_peak_TF_ and ventilatory parameters.

Therefore, the V_peak_TF_ showed high reliability in both TR and RR. These findings reinforce that the protocol for determining V_peak_TF_, using increments of 1 km⋅h^–1^ every 3 min is reliable regardless of the performance level of the runners.

## Practical Application

The test to determine the V_peak_TF_ can be considered a valid field test to estimate the MAS, and also to estimate submaximal and maximal HR and RPE values runners of different levels. These findings allow coaches to monitor and assess these runners’ aerobic performance using the V_peak_TF_. The results of the present study have important practical implications for coaches and runners for determining high intensity continuous and interval aerobic training loads and for the facility to evaluate multiple runners simultaneously.

## Data Availability Statement

The raw data supporting the conclusions of this article will be made available by the authors, without undue reservation.

## Ethics Statement

The studies involving human participants were reviewed and approved by University’s Human Research Ethics Committee. The patients/participants provided their written informed consent to participate in this study.

## Author Contributions

FMn and FMc: research concept and study design, literature review. FMn: data collection. FMn, CP, and FMc: statistical analyses, data analysis and interpretation, writing of the manuscript, or reviewing/editing a draft of the manuscript. All authors contributed to the article and approved the submitted version.

## Conflict of Interest

The authors declare that the research was conducted in the absence of any commercial or financial relationships that could be construed as a potential conflict of interest.

## Publisher’s Note

All claims expressed in this article are solely those of the authors and do not necessarily represent those of their affiliated organizations, or those of the publisher, the editors and the reviewers. Any product that may be evaluated in this article, or claim that may be made by its manufacturer, is not guaranteed or endorsed by the publisher.
