# Supplementary figures and images for: Reliability of Peak Running Velocity Obtained on the Track Field in Runners of Different Performance Levels
Source: Front Physiol. 2021 Dec 13;12:680913. doi: 10.3389/fphys.2021.680913 (PMC8710694; doi:10.3389/fphys.2021.680913)

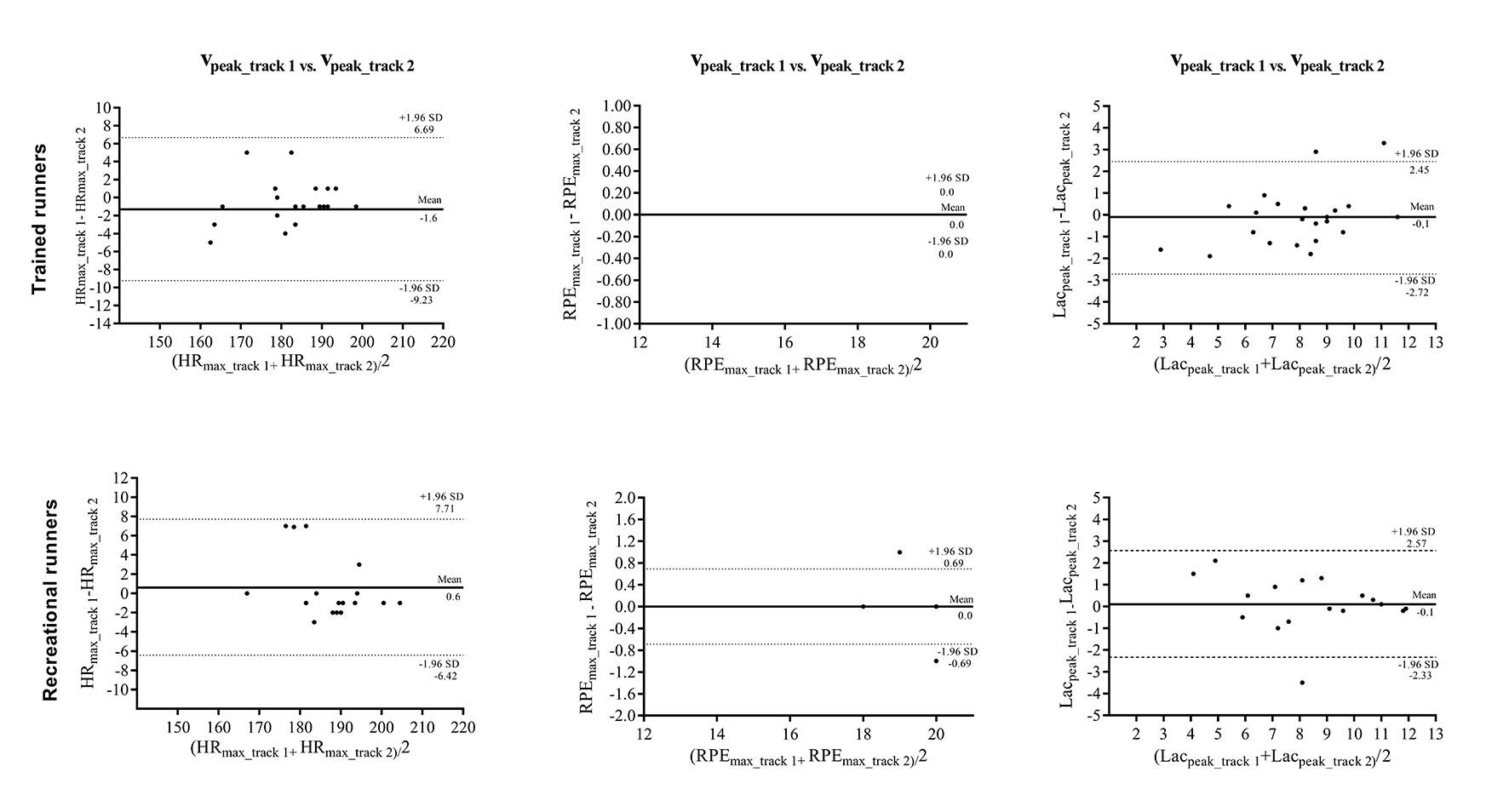

Supplement: Supplementary file 1 [file Image_1.TIF]

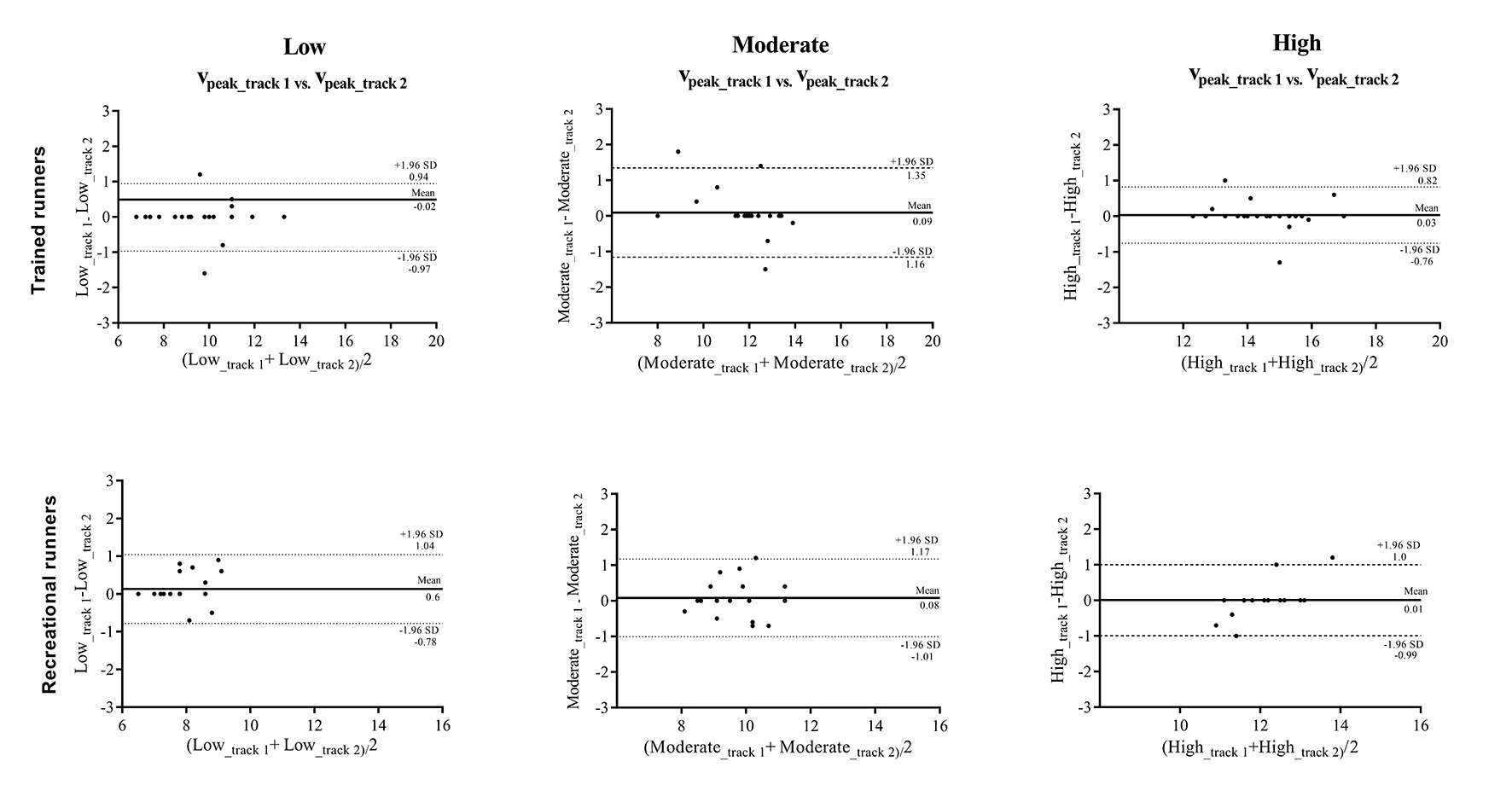

Supplement: Supplementary file 2 [file Image_2.TIF]
